# Nanomaterial accumulation in boiling brines enhances epithermal bonanzas

**DOI:** 10.1038/s41598-023-41756-4

**Published:** 2023-09-11

**Authors:** Néstor Cano, José M. González-Jiménez, Antoni Camprubí, Diego Domínguez-Carretero, Eduardo González-Partida, Joaquín A. Proenza

**Affiliations:** 1 Programa de Posgrado en Ciencias de la Tierra, Universidad Nacional Autónoma de México (UNAM). Ciudad Universitaria, 04510 Coyoacán, CDMX, Mexico; 2 Instituto Andaluz de Ciencias de la Tierra, CSIC-Universidad de Granada, Avda. de Las Palmeras 4, 18100 Armilla, Granada, Spain; 3 Instituto de Geología, UNAM. Ciudad Universitaria, 04510 Coyoacán, CDMX, Mexico; 4 Departament de Mineralogia, Petrologia i Geologia Aplicada, Facultat de Ciències de la Terra, Universitat de Barcelona, Martí I Franquès S/N, 08028 Barcelona, Spain; 5 Centro de Geociencias, UNAM, Blvd. Juriquilla 3001, 76230 Juriquilla, Qro. Mexico

**Keywords:** Mineralogy, Economic geology

## Abstract

Epithermal bonanza-type ores, characterized by weight-percent contents of e.g., gold and silver in a few mm to cm, are generated by mixtures of magmatic-derived hydrothermal brines and external fluids (e.g., meteoric) that transport a variety of metals to the site of deposition. However, the low solubilities of precious metals in hydrothermal fluids cannot justify the high concentrations necessary to produce such type of hyper-enriched metal ore. Here we show that boiling metal-bearing brines can produce, aggregate, and accumulate metal nanomaterials, ultimately leading to focused gold + silver ± copper over-enrichments. We found direct nano-scale evidence of nanoparticulate gold- and/or silver-bearing ores formed via nonclassical growth (i.e., nanomaterial attachment) during boiling in an intermediate-sulfidation epithermal bonanza. The documented processes may explain the generation of bonanzas in metal-rich brines from a range of mineral deposit types.

## Introduction

In the field of economic geology, nanomaterials may include atomic clusters, mineral nanoparticles (1–100 nm; NPs hereafter), nanominerals, nanofluids, and nanomelts, usually constituted by a wide suite of metals^1–3^. In the last decade, an ever-increasing amount of evidence has documented nanomaterials in hydrothermal solutions^4^, thus questioning the traditional paradigm that ligands (e.g., chloride- and bisulfide-bearing) are the sole efficient metal carriers in ore-forming fluids^5^. Indeed, metal complexes in solution at different physicochemical conditions account for metal endowments of ordinary ore deposits^5^. However, such a process does not fully explain precious metal over-enrichments (up to the wt%scale) in mm- to cm-sized bonanza-type ores^6, 7^.

Au–Ag ± Cu-bearing nanomaterials were documented in bonanzas from epithermal^4, 8, 9^ and orogenic gold deposits^7, 10^; both deposit types typically form by low-salinity ore-bearing fluids (< 10 wt%NaCl equiv.)^11, 12^. However, the actual mechanisms of transport, production, and deposition of these nanomaterials in hydrothermal fluids remain enigmatic, especially at moderate and high salinities. This is a consequence of the low preservation potential of the pristine nanofeatures of ore minerals, added to unconstrained physicochemical conditions of the causative fluids in previous nanoscale research on bonanzas.

This investigation focuses on the El Hilo bonanza from the Natividad intermediate sulfidation epithermal deposit in southern Mexico (longitude: 96.428° W, latitude: 17.303° N). In this region, magmatic pulses from the latest Oligocene–Miocene account for dozens of magmatic-hydrothermal ore deposits, most of them epithermal in type^13, 14^. We combined fluid inclusion studies, focused-ion beam (FIB) scanning electron microscopy (SEM), high-angle annular dark-field (HAADF) scanning transmission electron microscopy (STEM), and high-resolution transmission electron microscopy (HRTEM) to examine El Hilo, where nanoparticulate acanthite (AgS_2_), pearceite-polybasite ([Ag_9_CuS_4_][(Ag,Cu)_6_(As,Sb)_2_S_7_]), and electrum (AuAg) precipitated due to boiling of moderate-salinity hydrothermal fluids. These ore minerals comprise rounded non-oriented nanoparticles and close-packed clusters of nanoparticles dispersed in an amorphous matrix, suggesting nonclassical growth via nanomaterial attachment. Our observations suggest that boiling in hydrothermal ore-bearing brines can produce, aggregate, and accumulate metal nanomaterials that are seemingly responsible for the anomalously high metal contents in bonanza-type deposits.

## Results

**Figure 1:**
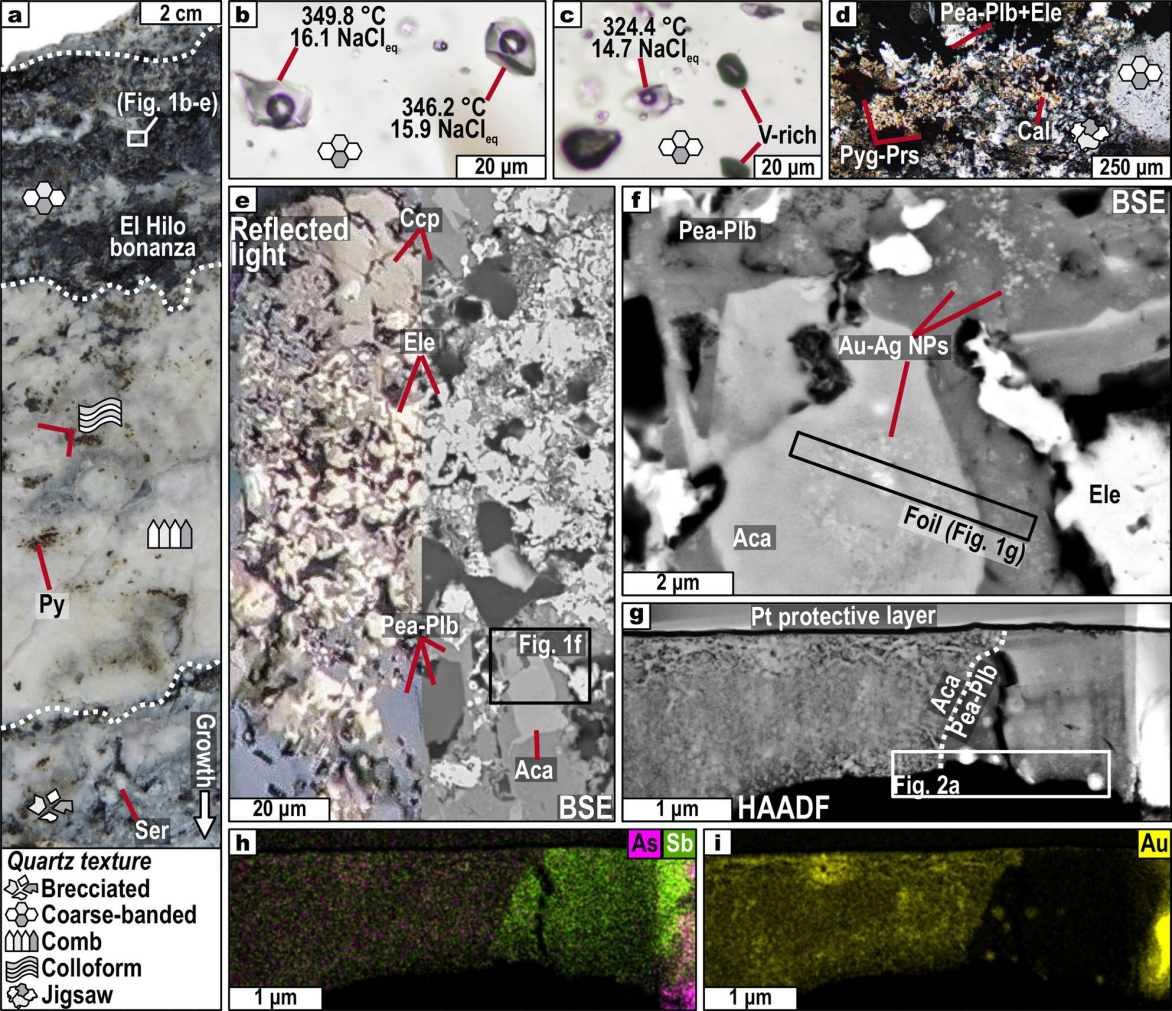
Macro, micro, and nanofeatures of the analyzed thin foil. (**a**) Banded texture of the Poder de Dios vein. (**b**), (**c**) Boiling-suggestive fluid inclusions in coarse-banded quartz coexisting with the ores. (**d**) Jigsaw and coarse-banded quartz with calcite, Ag-sulfosalts, and electrum. (**e**) Back-scattered electron (BSE) and reflected light images of the studied ore assemblage. (**f**) Closer view to Fig. (**e**) showing a BSE image of acanthite and pearceite-polybasite with electrum nanoparticles. The location of the thin foil is also portrayed. (**g**)–(**i**) High-angle annular dark-field (HAADF) image and compositional maps (As, Sb, and Au) of the thin foil. Key: Aca—acanthite, Cal—calcite, Ccp—chalcopyrite, Ele—electrum, Pea—pearceite, Plb—polybasite, Prs—proustite, Py—pyrite, Pyg—pyrargyrite, Ser—sericite.

The El Hilo bonanza is ~ 5 cm wide, attains the highest grades in the Natividad mine (up to 0.2 wt% Au and 3.1 wt% Ag), and is hosted by the Poder de Dios vein (Fig. 1a). This vein is one of the three structures that are currently mined at Natividad for Au and Ag, and it crosscuts low-grade Paleozoic metasedimentites^15, 16^. Petrographic observations of the bonanza zone show that jigsaw and coarse-banded quartz are the main gangue minerals, plus minor calcite and ankerite (Fig. 1b,c,d; Supplementary Figs. 2 and 3). Jigsaw quartz is interpreted to recrystallize from amorphous silica or gel precursors formed due to boiling^12, 17^. Coarse-banded quartz host coexisting liquid-rich and vapor-rich fluid inclusions (Fig. 1b,c)—also indicative of boiling—that yielded temperatures of homogenization at 273–397 °C and salinities of 13.9–18.7 wt% NaCl equiv. (Supplementary Table 1 and Supplementary Figs. 5 and 6).

The ores from El Hilo consist of early sulfide-dominated assemblages (pyrite + sphalerite + galena + chalcopyrite ± electrum), followed by sulfosalt-dominated assemblages (acanthite + pearceite-polybasite + electrum + pyrargyrite-proustite + aguilarite (Ag_4_SeS) + fahlore + pyrite + chalcopyrite; Fig. 1d,e). Ag-sulfosalts often replace galena, which contains electrum inclusions up to 50 µm in diameter, as well as electrum encapsulated in pyrite (Supplementary Fig. 3d,e). These observations reveal the presence of coupled dissolution-reprecipitation reactions^18^. As of electron probe micro-analyzer (EPMA) data, pearceite-polybasite has the empirical formula [Ag_16.0_Cu_1.5_Fe_0.2_Zn_0.1_As_1.0_Sb_1.1_S_11.0_Se_0.8_] and electrum yielded Au/(Au + Ag) ratios between 0.3 and 0.7 (Supplementary Tables 2 and 3). Moreover, using field-emission SEM we identified regions where acanthite and pearceite-polybasite contain nano-sized electrum particles (Fig. 1f,g,h,i). We prepared a thin foil from one of these regions by FIB-SEM (Supplementary Figs. 7–10) and analyzed it using HRTEM. Compositional mapping of the foil by STEM energy-dispersive X-ray spectroscopy (EDS) shows that Au is nearly homogeneously dispersed in acanthite, whereas it forms nano-sized clusters in pearceite-polybasite (Fig. 1h,i).

### Features of nanoparticulate Au–Ag–Cu ores

**Figure 2:**
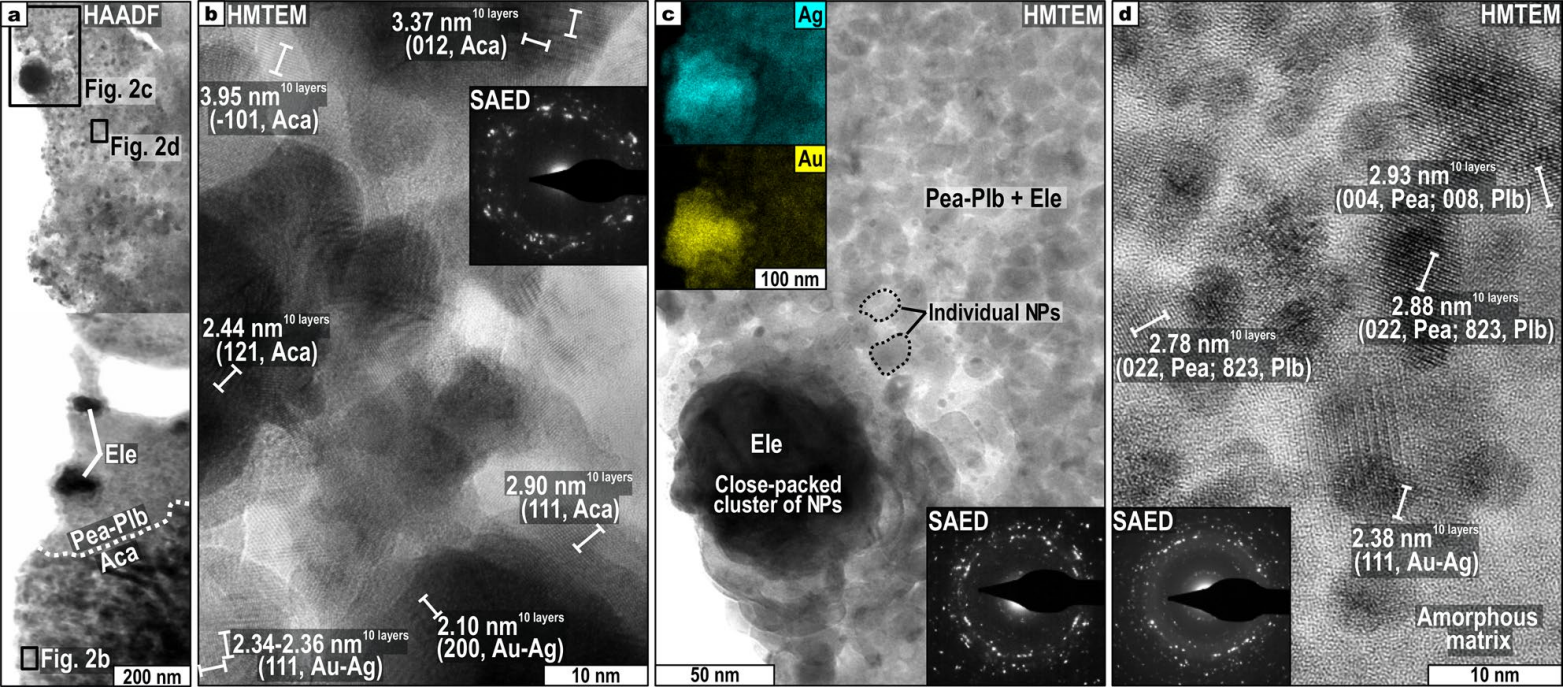
High-angle annular dark-field (HAADF), high-magnification transmission electron microscopy (HMTEM), and selected area electron diffraction (SAED) images of nanoparticulate ores. Measured *d-*spacings are also shown. (**a**) HAADF image of the contact between (1) acanthite and (2) pearceite-polybasite with electrum nanomaterials. (**b**) Randomly oriented crystalline nanoparticles (NPs) of acanthite and electrum. (**c**) Zoomed view to aggregates of pearceite-polybasite NPs surrounding close-packed clusters of electrum NPs. Au and Ag compositional maps are portrayed in insets. (**d**). Randomly oriented pearceite-polybasite and electrum NPs within an amorphous matrix. Mineral abbreviations as in Fig. 1.

High-magnification HRTEM imaging reveals that acanthite consists of aggregations of rounded-like crystalline NPs with sizes between ~ 10 and ~ 25 nm (Fig. 2a,b). The aggregations embed larger (up to ~ 30 nm) individual electrum NP, also rounded (Fig. 2b). These NPs show randomly oriented lattice planes in high magnification TEM, confirmed by concentric-like selected area electron diffraction (SAED) patterns (Fig. 2b). The lattice fringes measured in Fig. 2b have *d-*spacings similar to those of acanthite [e.g., ~ 2.90 Å, (111)] and gold and/or silver [e.g., ~ 2.34–2.36 Å, (111)].

Pearceite-polybasite also comprise mosaics of (1) rounded-like individual NPs of these minerals and electrum ranging from ~ 5 to ~ 15 nm, and (2) close-packed clusters of electrum NPs attaining up to 100 nm, all hosted in an amorphous matrix (Fig. 2c,d). Figure 2d displays that individual pearceite-polybasite and electrum NPs exhibit random lattice orientation with angular offsets, and usually overlap one another in cross-section. Consistently, the SAED diffractogram shows concentric patterns with varying radii (Fig. 2d). Likewise, concentric-like SAED patterns obtained in the closed-packed clusters reveal aggregated non-oriented electrum NPs (Fig. 2c). The *d-*spacings measured in Fig. 2d are similar to those of gold and silver [~ 2.38 Å, (111)], pearceite [e.g., ~ 2.78–2.88 Å, (022)], and polybasite [e.g., ~ 2.78–2.88 Å, (823)].

## Discussion

### *Bonanzas *via* boiling-induced nanomaterial accumulation in hydrothermal brines*

The ore minerals analyzed here are anhedral grains that lack dendritic textures and are made up of individual NPs or close-packed clusters of NPs dispersed in an amorphous matrix (Figs. 1 and 2). This suggests that the crystal matrices of such ore minerals are in reality a heterogenous mix of nanomaterials. In addition, the random arrangement of metal nanomaterials implies that crystal growth involved the aggregation of nanodomains instead of coarse crystal lattices. Thus, our results might represent the missing link between isolated metal nanomaterials and true crystals, and underpin a crystal growth mechanism via nonclassical nanomaterial attachment that differs from the “fractal” dendrites as of Saunders and Schoenly^19^.

Figures 1d,e show that phases rich in low-melting-point chalcophile elements (LMCE; e.g., Sb–As–Se-rich minerals) coexist with electrum. The round-shaped metal NPs in El Hilo and the calculated fluid temperatures up to 397 °C suggest the possibility that nanomaterials derived from LMCE-bearing nanomelts eventually formed as a result of boiling. This hypothesis is supported by experimental^20^ and thermodynamic^21, 22^ modeling, which confirms that LMCE-bearing phases melt at temperatures as low as ~ 300 °C and can depress Au(–Ag) melting point down to ~ 350 °C. In fact, Ag-rich electrum and kuestelite (Au-rich silver) nanotetrahedrons (~ 4 nm) can melt at temperatures as low as ~ 250 °C, provided that the melting point of Au–Ag-alloys is size- and shape-dependent^23, 24^.

**Figure 3:**
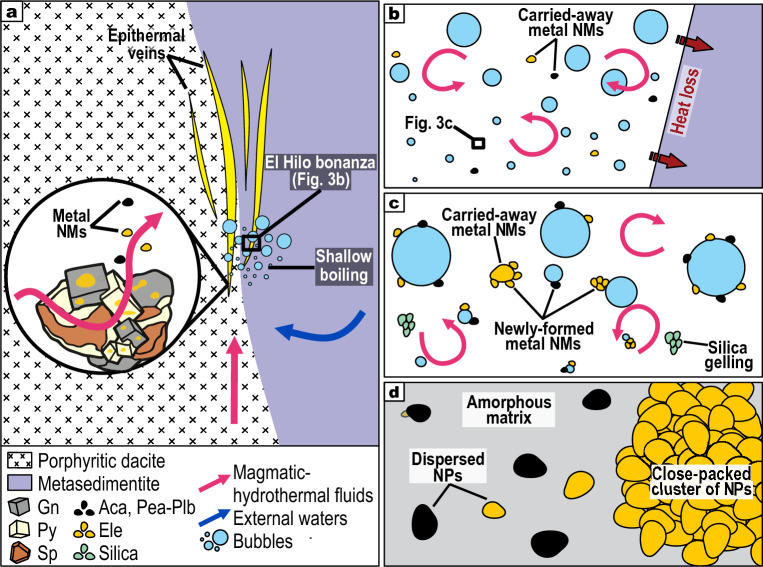
Schematic model for the formation of metal nanomaterials in the El Hilo bonanza. (**a**) Genetic model for the Natividad epithermal deposit showing a possible source for carried-away nanomaterials in the zoomed area. During ascent, deeply sourced magmatic-hydrothermal fluids can mix with external diluted waters. In the epithermal environment, these fluids react with sulfide(+ electrum) assemblages causing Au + Ag liberation as nanomaterials (zoomed area). (**b**), (**c**) Shallow boiling and heat loss of the brines induce metal supersaturation and heterogeneous seeding of nanomaterials at the surfaces of bubbles and/or earlier nanomaterials. This process is synchronous with silica gelling. The chaotic motion due to boiling allows nanomaterials to aggregate. (**a**) Individual newly-formed nanoparticles and close-packed clusters of newly-formed ± carried-away nanomaterials hosted in an amorphous matrix. Key: Aca—acanthite, Ele—electrum, Gn—galena, NM—nanomaterial, NP—nanoparticle, Pea-Plb—pearceite-polybasite, Py—pyrite, Sp—sphalerite.

Our temperature (273–397 °C) and salinity (13.9–18.7 wt% NaCl equiv.) data suggest that Au and Ag could have been initially transported in solution as Au(HS)_2_^–^ and AgCl_2_^–^^5^. Shallow boiling of upwelling brines coupled with conductive cooling (Fig. 3a,b), probably destabilized these complexes in solution, resulting in metal supersaturation and nanomaterial formation^5, 25^. The seeding of nanomaterials was likely heterogeneous^25^, as one could expect in boiling hydrothermal fluids having numerous nucleation sites. Therefore, acanthite, pearceite-polybasite, and electrum seeds could have developed at the surfaces of bubbles or nanomaterials suspended in the boiling solution (Fig. 3c). Meanwhile, boiling promoted silica supersaturation, gelling, and coagulation^17^, all of these processes accelerated by moderate salinities^26^.

After the seeding process, boiling enhanced the Brownian motion of newly-formed metal nanomaterials, thus overcoming surface-charge repulsions, leading to their ingrowth via aggregation (orthokinetic colloid aggregation)^27^ and subsequent accumulation to form the bonanza-type ores (Fig. 3c,d). Given that close-packed clusters of electrum NPs are up to 100 nm, exceeding the ranges of individual electrum NPs (5–15 nm; Fig. 2d), it is likely that their aggregation process occurred at the expense of earlier nanomaterials, either newly-formed or carried-away nanomaterials (Fig. 3c,d). Once deposited, solid-state non-oriented attachment (e.g., Ostwald ripening)^28^ probably led to the equigranularity observed in individual NPs (Fig. 2d).

Fluid unmixing phenomena (boiling, effervescence, or flashing/vaporization) of metal-rich hydrothermal brines, like those studied herein (Supplementary Fig. 6), may occur in e.g., volcanogenic massive sulfide, metalliferous porphyry, skarn, and intrusion-related gold deposits^5, 29^. Therefore, our nanomaterial-based model could be applied to ore-bearing brines from different geological contexts in order to explain the development of focused precious metal over-enriched zones.

### Nanomaterial persistence and preservation in hydrothermal ore systems

The dominant texture at the El Hilo bonanza is massive while no colloform-banded texture occurs, suggesting that it records a single Au–Ag–Cu-rich magmatic-hydrothermal pulse (Fig. 1a). Hence, we can discard the chance of episodic replenishment with juvenile magmatic components, which is a process that seems to constrain the formation of low-sulfidation epithermal bonanzas^30, 31^. The prevalence of moderate-salinity brines (up to. 18.7 wt% NaCl equiv.) in the epithermal environment is suggestive of some magmatic affinity by the entrainment of magmatic-derived brines^32, 33^ (Fig. 3a), probably mixed with diluted fluids (i.e., deeply circulated meteoric water, condensed magmatic vapor, or late low-salinity magmatic fluid)^12, 34^.

The occurrence of coupled dissolution-reprecipitation reactions (CDRR) affecting the electrum-bearing sulfide-dominated assemblages provides a likely source for carried-away nanomaterials (Fig. 3a). CDRR probably liberated Au ± Ag ± Cu to the fluids, where fluid-mediated LMCE-bearing nanomelts could have scavenged metals and transported them to the site of shallow boiling. This possibility agrees with previous models applied to orogenic gold and intrusion-related gold deposits^21, 35^, thus suggesting that it would be a more common process than previously thought. Alternatively, the earliest carried-away nanomaterials could have seeded and grown at depth (i.e., magmatic environment) and then mechanically transported upwards by rising magmatic-hydrothermal brines. During their ascension, the electrostatic repulsion between negatively charged nanomaterials would have kept them suspended in the solution^36^.

We propose that concomitant boiling and conductive cooling froze in time the observed NP aggregates by cementing them with the amorphous compound (Fig. 2d). Given that quartz is not well-crystallized nor contains deformed fluid inclusions, we presume that late deformation and/or recrystallization were mild, if any. Altogether, the serendipitous convergence of these factors can explain the outstanding preservation of the nanoscale features in El Hilo.

## Methods

### Petrography and scanning electron microscopy (SEM)

Descriptions of ore and gangue assemblages were completed at the Instituto de Geología, UNAM. After selecting areas of interest, acanthite and pearceite-polybasite hosting Au–Ag nanoparticles were characterized and imaged using a JEOL J-7100 field emission SEM at the Centres Científics i Tecnològics (Universitat de Barcelona), Spain. The instrument is equipped with an energy dispersive spectra (EDS) detector. Accelerating voltage was 20 kV and beam current optimized for an adequate number of counts for each EDS analysis.

### EPMA analyses

Mineral chemistries were studied with a JEOL JXA-8230 electron probe micro-analyzer located at the Centres Científics i Tecnòlogics (Universitat de Barcelona). The apparatus was operated at 20 kV acceleration voltage, 20 nA beam current, with a beam diameter of 1 μm. Analytical standards and additional conditions are specified in Supplementary Table 1, and the results are summarized in Supplementary Table 3.

### Focused-ion beam (FIB)-SEM analyses

The thin foil was prepared using a Dual Beam FEI Thermo-Fisher Scientific Helios 650 FIB-SEM at the Laboratorio de Microscopías Avanzadas at the Instituto de Nanociencia de Aragón (Supplementary Fig. 7), following procedures described by González-Jiménez et al^1^. The area containing Au–Ag nanoparticles was covered with C (~ 300 nm) and Pt (~ 1 μm) layers to protect it during the milling, polishing, and extraction process. The bulk material was removed on both sides of the thin foil by Ga + ion milling at 30 kV and 2.5 nA. The thin foil was then extracted from the sample and transferred to a TEM Cu grid using an OmniProbe nanomanipulator with a tungsten tip, where the thinning process was performed with the Ga + ion beam at 5–30 kV and 68 pA–0.23 nA. After achieving the electron transparency (~ 90 nm), the thin foil was polished using a low energy 5 kV current at 10 pA to reduce amorphization.

### High-resolution transmission electron microscopy (HRTEM) analyses

The thin foil was analyzed with a FEI Titan G2 HRTEM equipped with Field Emission gun XFEG, running at 300 kV. The apparatus is available at the Centro de Instrumentation Científica of the University of Granada, Spain. Compositional maps were obtained by energy-dispersive X-ray spectroscopy (EDS) using the Super-X system and were processed with the VELOX® software package. High-angle annular dark-field scanning electron microscopy (HAADF-STEM) and high-magnification HRTEM were used to describe nanoparticles and to index minerals. Selected area electron diffraction (SAED) and fast-Fourier transform (FFT) diffractograms of the interest areas were employed to confirm the presence of nanoparticles. The images were treated using the Digital Micrograph® software in its Version 1.71.38.

### Fluid inclusion studies

Fluid inclusion petrography and microthermometric studies were conducted in coarse-banded quartz crystals in spatial association with the ores in the El Hilo bonanza. Individual quartz grains are < 600 µm, display uniform and plumose extinction, and do not have evidence of recrystallization, according to criteria by Sander and Black^37^ and Dong et al^17^. In addition, the arrangement of fluid inclusions does not insinuate an inherited character from chalcedony precursors^37^. Hence, they were considered actual aliquots of the mineralizing fluids. We analyzed fluid inclusions from isolated clusters and healed fractures, with no apparent post-trapping modifications. For petrographic descriptions of fluid inclusions, FIA (fluid inclusion assemblage) definition, and classification as primary, secondary, or pseudo-secondary, we followed Roedder^38^, Goldstein and Reynolds^39^, and Van Den Kerkhof and Hein^40^. Microthermometry was performed with a petrographic microscope Olympus BX60 coupled with a Linkam THMSG 600 stage, available at the Laboratorio de Catodoluminiscencia e Inclusiones Fluidas, UNAM. The stage operates in the range from − 200 to 600 °C. Estimated accuracy is ± 0.2 °C low temperature essays, and within ± 2 °C for homogenization temperatures. Data processing was performed with HokieFlincs_H2O–NaCl^41^, using ice melting temperature to calculate salinity. The results are summarized in Supplementary Table 1.

### Supplementary Information

Supplementary Information 1.

Supplementary Table 1.

## Data Availability

All analyzed data for this study are provided in the article and Supplementary Information.
