# Supplementary material for: Nanomaterial accumulation in boiling brines enhances epithermal bonanzas
Source: Sci Rep. 2023 Sep 11;13:14985. doi: 10.1038/s41598-023-41756-4 (PMC10495403; doi:10.1038/s41598-023-41756-4)
Supplement: Supplementary file 1 — Supplementary Information 1. [file 41598_2023_41756_MOESM1_ESM.pdf]

Supplementary Information for:

Nanomaterial accumulation in boiling brines enhances epithermal bonanzas

Néstor Cano[1]*, José M. González-Jiménez[2], Antoni Camprubí[3], Joaquín Proenza[4], Eduardo González-Partida[5], and Diego Domínguez-Carretero[4]

[1]Programa de Posgrado en Ciencias de la Tierra, Universidad Nacional Autónoma de México (UNAM). Ciudad Universitaria, 04510 Coyoacán, CDMX, Mexico

[2]Instituto Andaluz de Ciencias de la Tierra, CSIC-Universidad de Granada, Avda. de las Palmeras 4, 18100 Armilla, Granada, Spain

[3]Instituto de Geología, UNAM. Ciudad Universitaria, 04510 Coyoacán, CDMX, Mexico

[4]Departament de Mineralogia, Petrologia i Geologia Aplicada, Facultat de Ciències de la Terra, Universitat de Barcelona, Matí i Franquès s/n, 08028 Barcelona, Spain

[5]Centro de Geociencias, UNAM. Blvd. Juriquilla 3001, 76230 Juriquilla, Qro., Mexico

*Corresponding author (NC): nacanoh@comunidad.unam.mx

The Supplementary Information contains:

1. **SUPPLEMENTARY FIGURES**
   1.1 Supplementary Figure 1 Geological map of the Natividad mining district
   1.2 Supplementary Figure 2 Hand sample of the Poder de Dios vein and closer view to the El Hilo bonanza
   1.3 Supplementary Figure 3 Photomicrographs of the El Hilo bonanza
   1.4 Supplementary Figure 4 Paragenetic sequence of the El Hilo bonanza
   1.5 Supplementary Figure 5 Fluid inclusion assemblages hosted by coarse-banded quartz associated with the ores at El Hilo
   1.6 Supplementary Figure 6 Salinity vs. homogenization temperature plot
   1.7 Supplementary Figure 7 Thin foil preparation using focus ion-beam scanning electron microscopy FIB-SEM
   1.8 Supplementary Figure 8 Back-scattered electron image of the studied sulfide + sulfosalt + electrum cluster and energy dispersive spectroscopy (EDS) spectra
   1.9 Supplementary Figure 9 Thin foil and scanning transmission electron microscopy EDS spectra
   1.10 Supplementary Figure 10 Compositional maps of the contact between acanthite and pearceite-polybasite in the studied foil
   1.11 Supplementary Figure 11 High-magnification TEM (HMTEM) images of nanoparticulate acanthite, pearceite-polybasite, and electrum
   1.12 Supplementary Figure 12 Selected area electron diffraction patterns
   1.13 Supplementary Figure 13 HAADF, HMTEM, and SAED images of individual and close-packed clusters of electrum NPs

2. **TABLES**
   2.1 Supplementary Table 1 Fluid inclusion petrography and microthermometry results
   2.2 Supplementary Table 2 Electron probe microanalysis (EPMA) standards
   2.3 Supplementary Table 3 EPMA results

3. **SUPPLEMENTARY REFERENCES**

1. SUPPLEMENTARY FIGURES

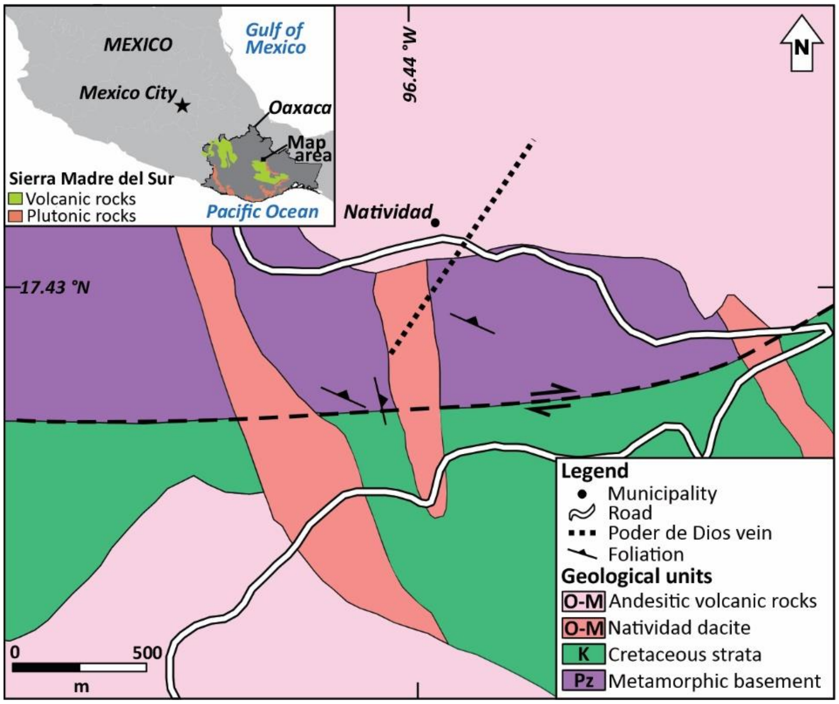

Supplementary Figure 1. Geological map of the study zone (Datum: WGS94). Upper left inset exhibits the distribution of volcanic and plutonic rocks of the Sierra Madre del Sur igneous province in the state of Oaxaca[1]. Modified from SGM[2,3], based on fieldwork. Ages based on stratigraphic position, according to SGM[2,3]. Pz: Paleozoic, K: Cretaceous, O-M: Oligocene-Miocene.

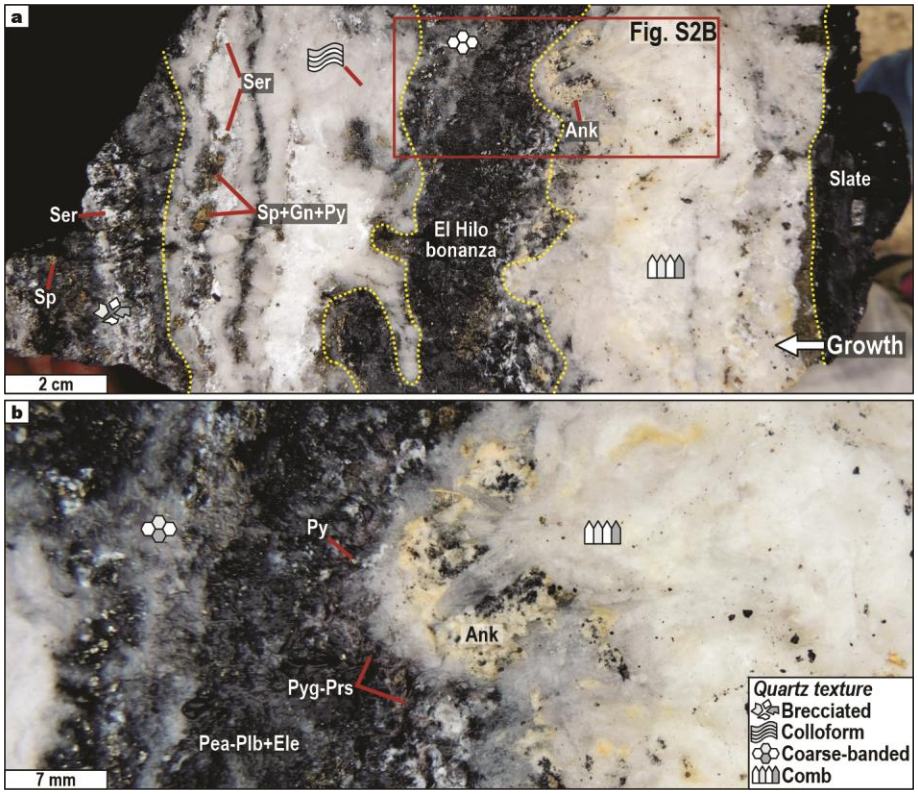

Supplementary Figure 2. Hand sample of the Poder de Dios vein (A) and closer view to the El Hilo bonanza (B). The quartz ± carbonate vein displays crustiform (asymmetric)-banded structure that consist of, from the wall-rock inwards (growth direction): 1) comb quartz with scarce ore minerals; 2) El Hilo bonanza; 3) colloform and saccharoidal quartz with sericite + sphalerite + galena + pyrite bands and patches; and 4) hydraulic breccia with patches of sericite and sphalerite. Notice in B. proustite-pyrargyrite (red patches) and pearceite-polybasite + electrum + sulfide (black region) coexisting with coarse-banded quartz, as well as ankerite + comb quartz. Mineral abbreviations: Ank = ankerite, Ele = electrum, Gn = galena, Pea = pearceite, Plb = polybasite, Prs = proustite, Py = pyrite, Pyg = Pyrargyrite, Ser = sericite, Sp = sphalerite.

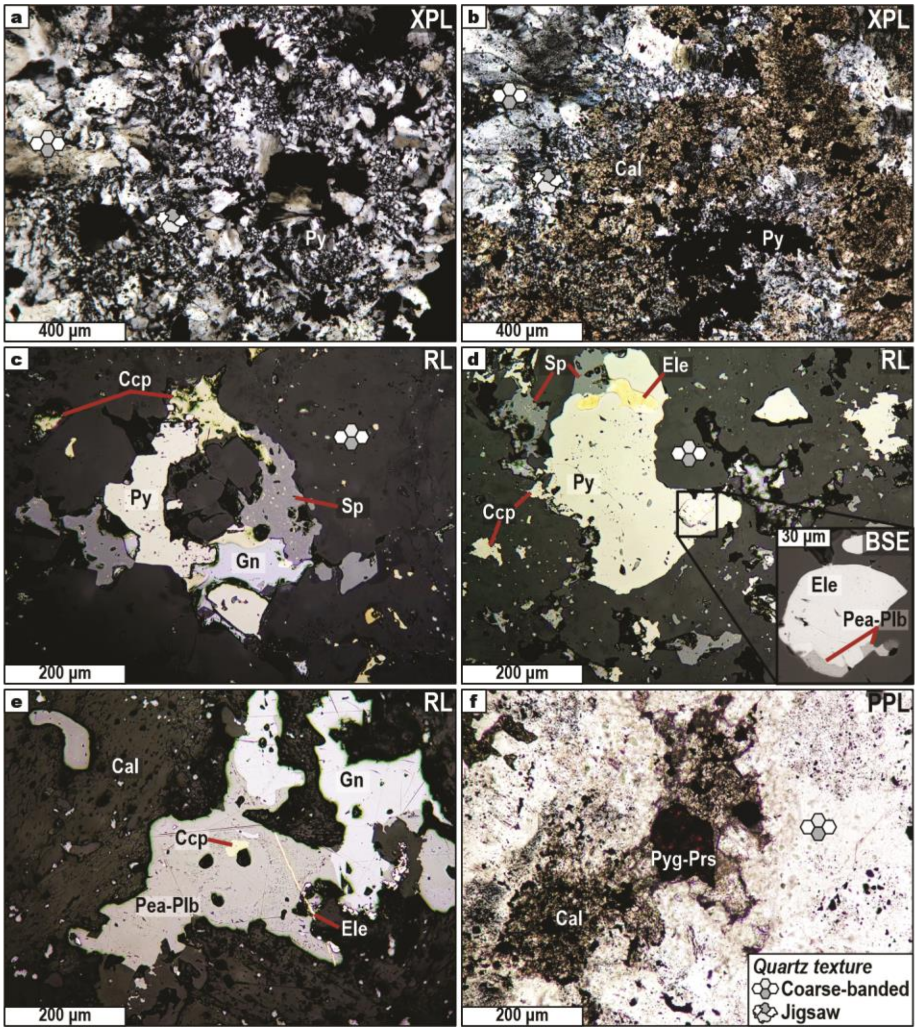

Supplementary Figure 3. Photomicrographs of the El Hilo bonanza. A-B. Jigsaw and coarse-banded quartz coexisting with calcite and pyrite. C-D. Ore assemblage of the sulfide-dominated stage (stage 1 in Supplementary Figure 4), consisting of galena, pyrite, chalcopyrite, sphalerite (chalcopyrite disease), and electrum inclusions. Inset in Supplementary Figure 3D depicts electrum replaced by pearceite-polybasite. E. Galena partly replaced by pearceite-polybasite from the sulfosalt-dominated assemblage (stage 2 in Supplementary Figure 4). Notice an electrum stringer crosscutting the sulfosalt. F. Pyrargyrite-proustite associated with calcite and coarse-banded quartz. Image type: BSE = back-scattered electron, PPL = plane-polarized light, XPL = cross-polarized light, RL = reflected light. Mineral abbreviations: Cal = calcite, Ccp = chalcopyrite, Ele = electrum, Gn = galena, Pea = pearceite, Plb = polybasite, Prs = proustite, Py = pyrite, Pyg = Pyrargyrite, Sp = sphalerite.

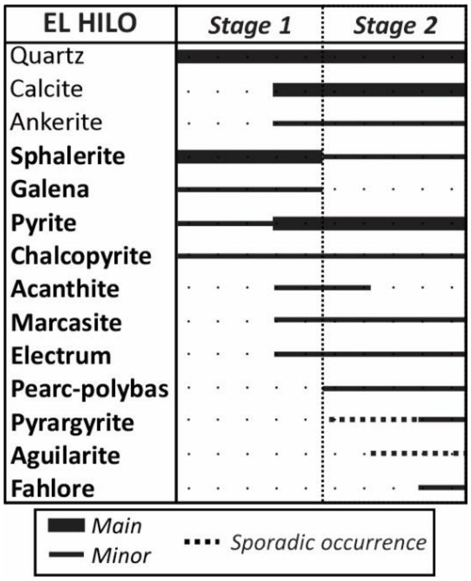

Supplementary Figure 4. Paragenetic sequence of the El Hilo bonanza. The stages 1 and 2 are the sulfide- and sulfosalt-dominated assemblages, respectively. Pearc-Polybas = pearceite-polybasite.

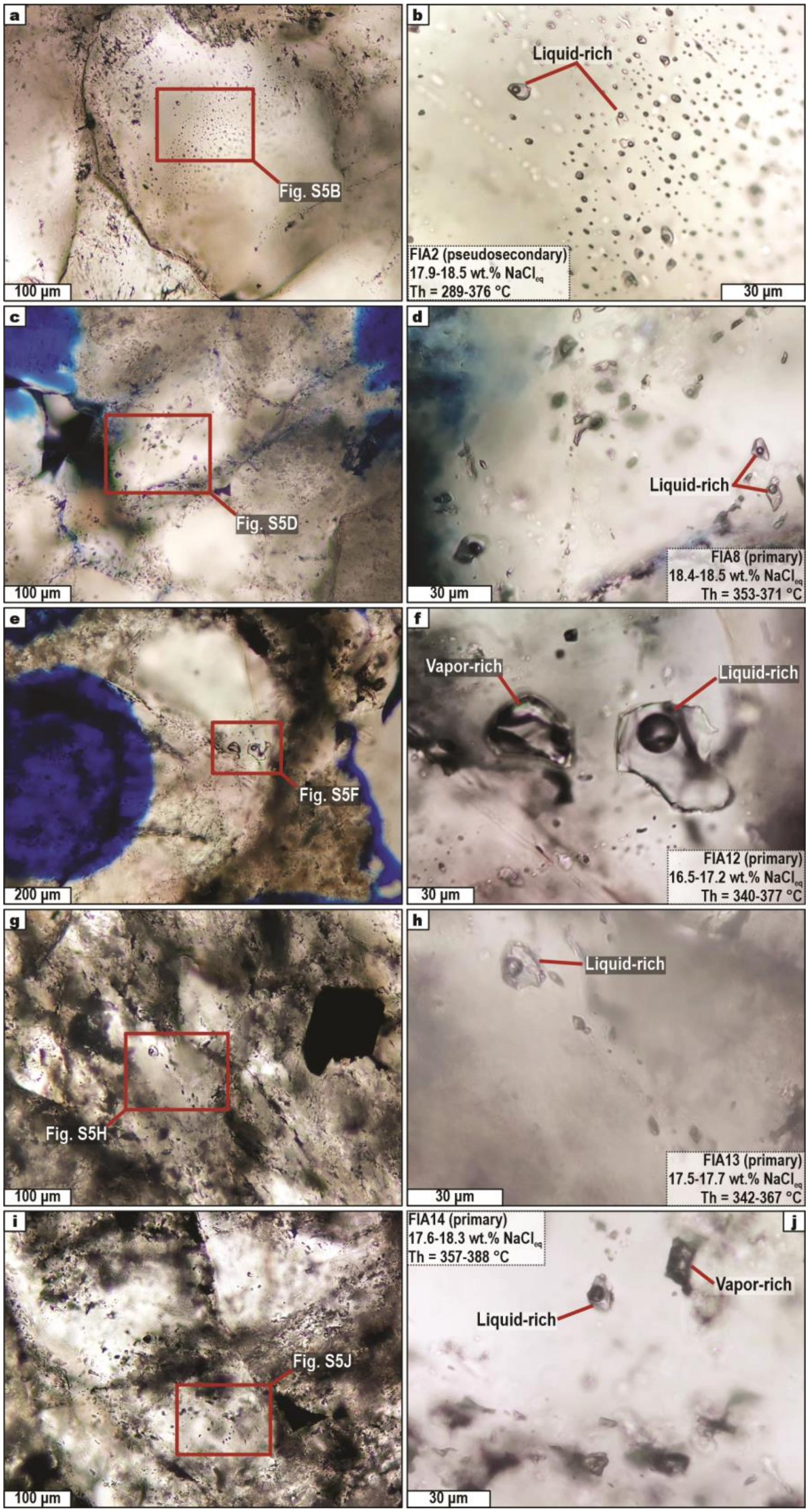

Supplementary Figure 5. Fluid inclusion assemblages (FIAs) hosted by coarse-banded quartz associated with the ores at El Hilo. We include the number of the FIA as in **Table 1**, type (primary, secondary, or pseudosecondary), and salinity and homogenization temperature ranges.

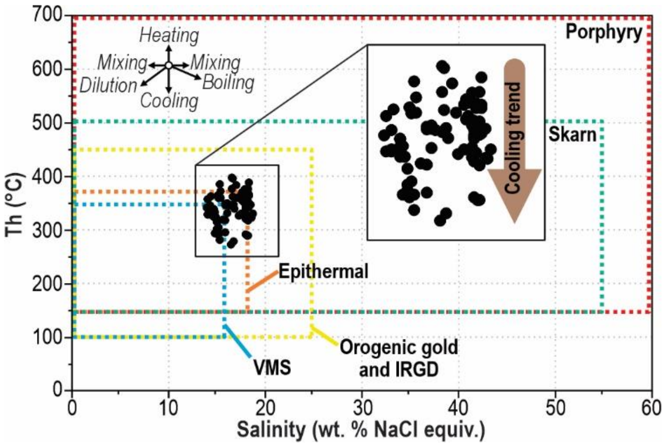

Supplementary Figure 6. Salinity vs. *Th* (temperature of homogenization) in fluid inclusions from the El Hilo bonanza (solid circles). A closer view to the data is shown in inset, where a cooling trend is drawn. Typical ranges for fluid inclusions from different deposit types are portrayed[4], as well as fluid evolutionary trends as of Wilkinson[5]. IRGD: intrusion-related gold deposits.

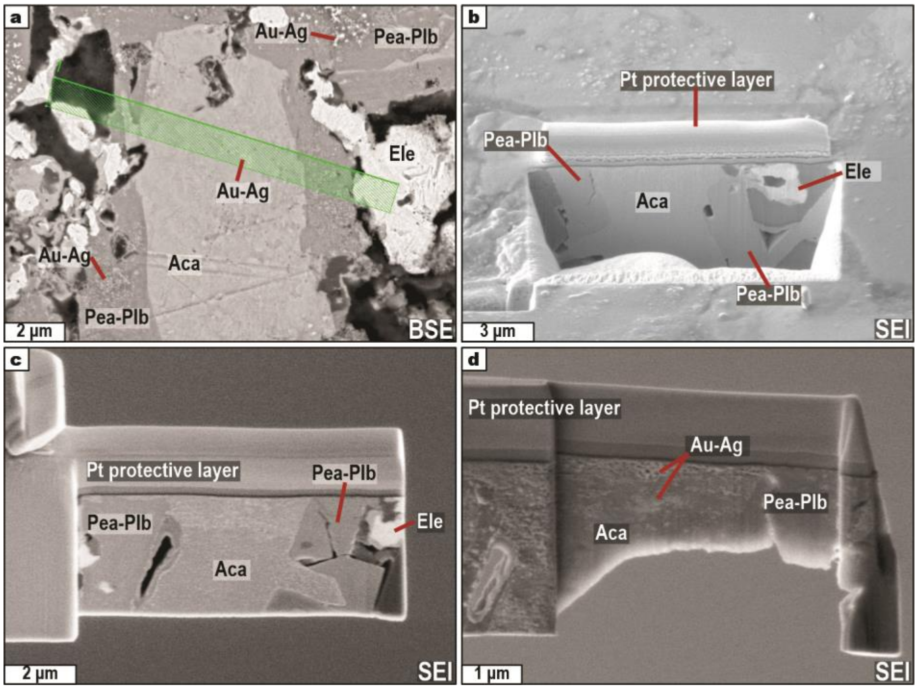

Supplementary Figure 7. Thin foil preparation using FIB-SEM. A. Back-scattered electron (BSE) image of the sampled area, comprising acanthite and pearceite-polybasite with electrum nanoparticles (green area). B-C. Secondary-electron image (SEI) showing pre-milled cross sections of the sampled foil. D. SEI image of the thin foil analyzed with HRTEM. Image type. SEI = Mineral abbreviations as in Supplementary Figure 3.

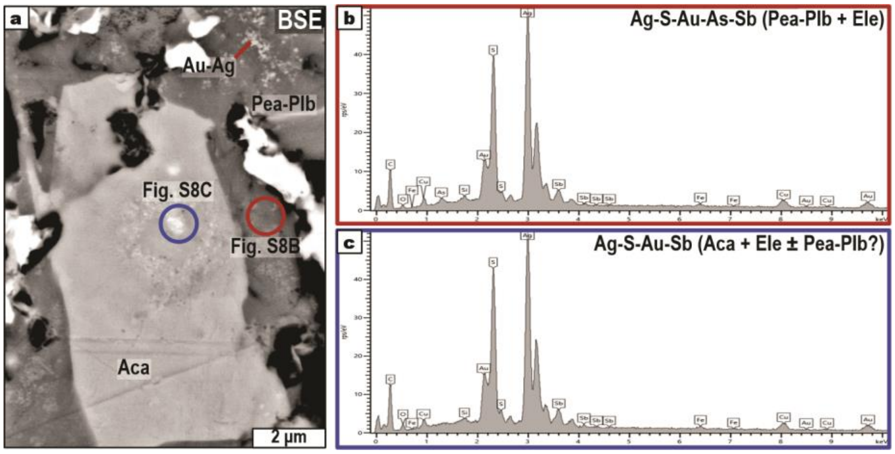

Supplementary Figure 8. BSE image of the acanthite + pearceite-polybasite + electrum assemblage (A) and energy dispersive spectroscopy (EDS) spectra (B-C). Mineral abbreviations as in Supplementary Figure 3.

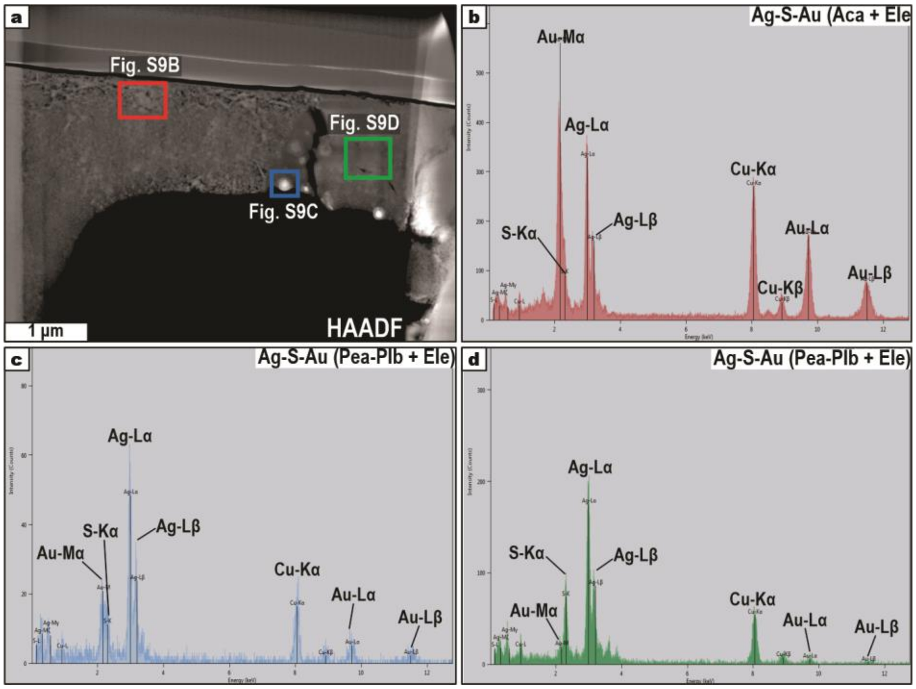

Supplementary Figure 9. High-angle annular dark-field (HAADF) image of the thin foil (A) and scanning transmission electron microscopy (STEM)-EDS spectra (B-D). Mineral abbreviations as in Supplementary Figure 3.

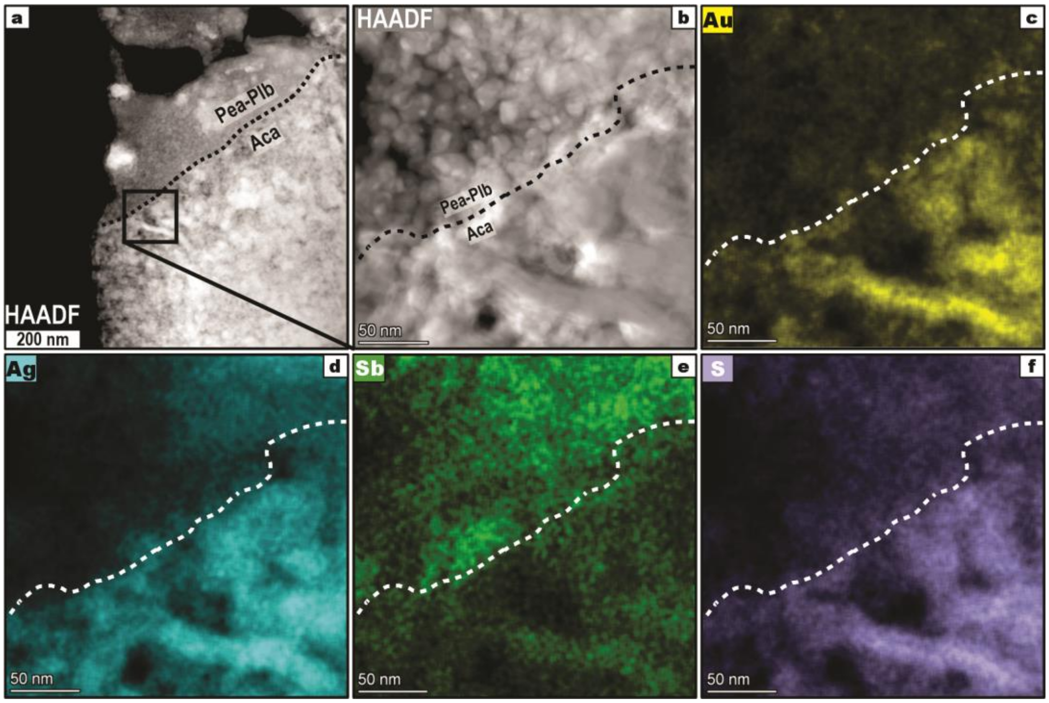

Supplementary Figure 10. HAADF images and STEM compositional maps of the contact between acanthite and pearceite-polybasite. Mineral abbreviations as in Supplementary Figure3.

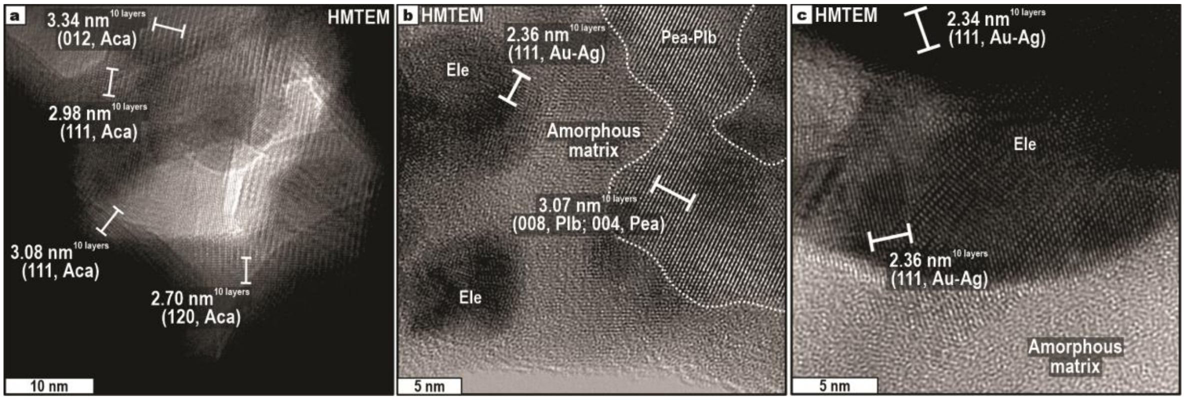

Supplementary Figure 11. High-magnification TEM (HMTEM) images showing aggregations of nanoparticles of acanthite (A), pearceite-polybasite + electrum (B), and electrum (C). Measured *d*-spacings are also presented. Notice the amorphous matrix that surrounds electrum and pearceite-polybasite NPs in B and C. Mineral abbreviations as in Supplementary Figure 3.

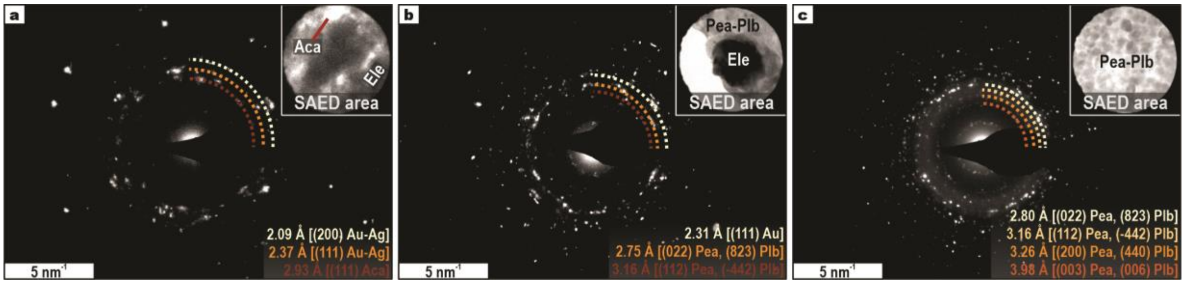

Supplementary Figure 12. Selected area electron diffraction (SAED) showing concentric-like patterns that are suggestive of randomly oriented NPs of acanthite + electrum (A), Pea-Plb + electrum (B), and Pea-Plb (C). Measured *d*-spacings are also provided. Abbreviations as in Supplementary Figure 3.

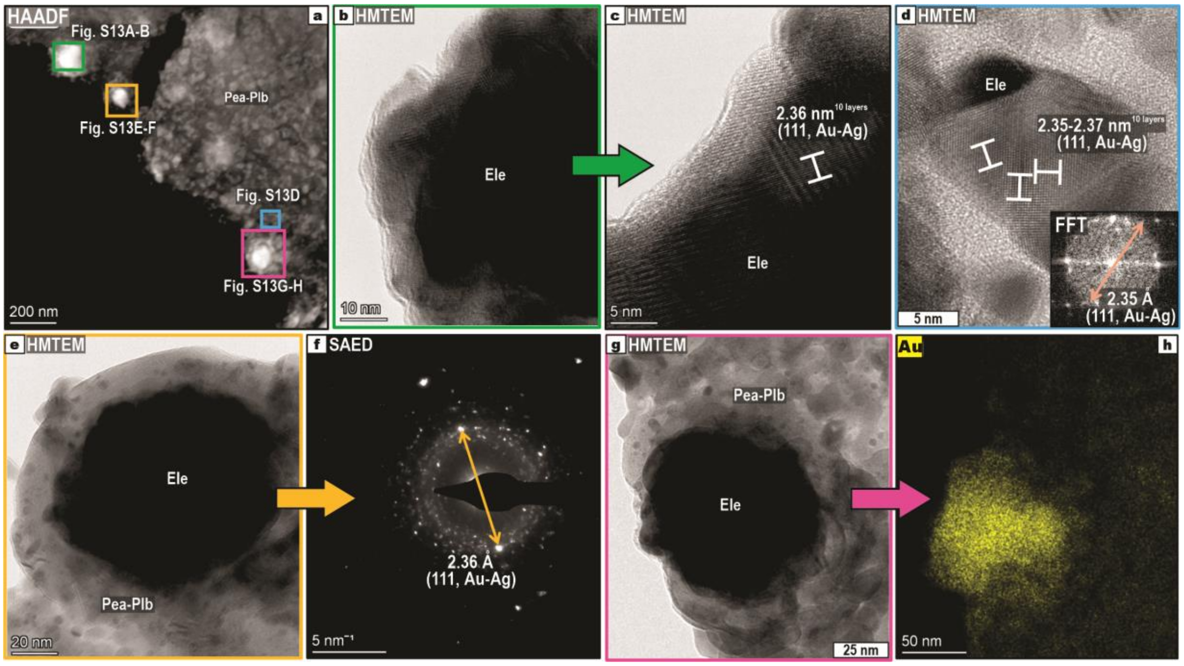

Supplementary Figure 13. HAADF, HMTEM, and SAED images of individual and close-packed clusters of electrum NPs. A. HAADF image of pearceite-polybasite with electrum nanomaterials. B-C. HMTEM images of the green square in Supplementary Figure 13A, with measured *d*-spacings. D. HMTEM image and fast Fourier transform (FFT; lower right inset) diffractogram of an individual electrum NP (blue square in Supplementary Figure 13A). E-F. HMTEM and SAED images for the orange square in Supplementary Figure 13A. G-H. HMTEM image and Au compositional map for the pink square in Figure 13A. Mineral abbreviations as in Supplementary Figure 3.

2. SUPPLEMENTARY TABLES

Supplementary Table 1 (Excel file). Fluid inclusion petrography and microthermometry results.

Supplementary Table 2. Electro probe micro-analyzer (EPMA) standards.

Element/ X-ray	X-ray	Crystal	Peak position	Standard	Composition
			Electrum		
Ag	La	PETJ	132.765	Ag	Ag (100.00 wt.%)
Fe	Ka	LIFH	134.658	Chalcopyrite	Fe (30.43 wt.%), Cu (34.63 wt.%), S (34.94 wt.%)
Cu	Ka	LIFH	107.06	Chalcopyrite	Fe (30.43 wt.%), Cu (34.63 wt.%), S (34.94 wt.%)
Au	Ma	PETL	187.067	Au	Au (100 wt.%)
S	Ka	PETL	172.083	Chalcopyrite	Fe (30.43 wt.%), Cu (34.63 wt.%), S (34.94 wt.%)
			Pyrargyrite-proustite		
Ag	La	PETJ	132.752	Ag	Ag (100.00 wt.%)
Sb	La	PETJ	109.681	Sb	Sb (100 wt.%)
Pb	Ma	PETJ	168.93	PbS	Pb (86.6%), S (13.4 wt.%)
Se	La	TAPH	97.592	In2Se	In (49.2 wt.%), Se (50.8 wt.%)
As	Lb	TAPH	105.022	GaAs	Ga (48.2 wt.%), As (51.8 wt.%)
Ge	Lb	TAPH	110.513	Ge	Ge (100 wt.%)
Fe	Ka	LIFH	134.658	Chalcopyrite	Fe (30.43 wt.%), Cu (34.63 wt.%), S (34.94 wt.%)
Cu	Ka	LIFH	107.06	Chalcopyrite	Fe (30.43 wt.%), Cu (34.63 wt.%), S (34.94 wt.%)
Zn	Ka	LIFH	99.644	Sphalerite	Zn (67.1 wt.%), S (32.9 wt.%)
S	Ka	PETL	172.098	Sphalerite	Zn (67.1 wt.%), S (32.9 wt.%)
Au	Ma	PETL	187.067	Au	Au (100 wt.%)

Supplementary Table 3. EPMA results.

		**Electrum**			
		**Spot 1**	**Spot 2**	**Spot 3**	**Spot 4**
**wt. %**	**Ag**	47.04	21.64	20.50	24.05
	**Fe**	0.22	0.38	0.39	0.24
	**Cu**	0.00	0.50	0.48	0.16
	**Au**	52.73	77.33	77.40	72.65
	**S**	0.20	0.59	0.11	0.05
	**Total**	100.19	100.45	98.88	97.17
**at. %**	**Au**	0.20	0.39	0.39	0.25
	**Ag**	0.46	0.20	0.19	0.29
**Au/(Au+Ag)**		0.37	0.66	0.67	0.62

		Spot 1	Spot 2	Spot 3	Spot 4	Spot 5	Spot 6	Spot 7
**Pearceite-polybasite**								
**wt. %**	**Sb**	3.50	4.20	5.83	6.09	5.95	4.08	5.40
	**Ag**	66.40	67.76	69.51	66.58	66.11	69.87	67.99
	**Pb**	0.03	0.02	0.05	0.16	0.12	0.00	0.09
	**Se**	2.13	2.42	2.58	3.20	2.78	2.16	2.80
	**As**	3.12	3.30	2.21	2.37	2.63	3.50	2.68
	**Ge**	0.00	0.33	0.00	0.00	0.00	0.01	0.00
	**Fe**	0.16	0.21	0.52	0.38	0.54	0.56	0.46
	**Cu**	3.90	3.85	3.72	3.51	3.32	4.22	3.63
	**Zn**	0.15	0.13	0.09	0.14	0.12	1.31	0.18
	**S**	14.66	14.69	13.54	13.76	13.39	13.34	13.61
	**Au**	1.95	1.08	1.34	3.38	3.71	1.02	2.94
	**Total**	96.01	97.98	99.40	99.57	98.67	100.07	99.78
**at. %**	**Ag**	49.38	49.39	51.44	49.39	49.74	50.88	50.29
	**Sb**	2.31	2.71	3.82	4.00	3.97	2.63	3.54
	**Se**	2.16	2.41	2.61	3.24	2.86	2.15	2.83
	**As**	3.34	3.46	2.35	2.53	2.85	3.67	2.85
	**Ge**	0.00	0.37	0.00	0.00	0.00	0.01	0.00
	**Fe**	0.23	0.30	0.75	0.54	0.78	0.79	0.66
	**Cu**	4.92	4.76	4.67	4.42	4.24	5.22	4.56
	**Zn**	0.19	0.16	0.11	0.17	0.15	1.57	0.22
	**S**	36.68	36.02	33.71	34.34	33.89	32.68	33.86
	**Au**	0.79	0.43	0.54	1.37	1.52	0.40	1.18
**Apfu***	**Ag**	14.81	15.08	16.79	15.82	16.14	17.13	16.33
	**Sb**	0.69	0.83	1.25	1.28	1.29	0.89	1.15
	**Se**	0.65	0.74	0.85	1.04	0.93	0.72	0.92
	**As**	1.00	1.06	0.77	0.81	0.92	1.24	0.93
	**Ge**	0.00	0.11	0.00	0.00	0.00	0.00	0.00
	**Fe**	0.07	0.09	0.24	0.17	0.25	0.26	0.22
	**Cu**	1.48	1.45	1.52	1.42	1.38	1.76	1.48
	**Zn**	0.06	0.05	0.04	0.06	0.05	0.53	0.07
	**S**	11.00	11.00	11.00	11.00	11.00	11.00	11.00

*Atoms per formula unit (apfu) calculated based on 11 atoms of S

3. SUPPLEMENTARY REFERENCES

1. Morán-Zenteno, D. J. et al. Cenozoic magmatism of the Sierra Madre del Sur and tectonic truncation of the Pacific margin of southern Mexico: Earth-Sci. Rev. **183**, 85–114; https://doi.org/10.1016/j.earscirev.2017.01.010 (2018).
2. Servicio Geológico Mexicano (SGM). Carta geológico-minera Santa Catarina Ixtepeji E14-D38. Scale 1:50,000, 1 sheet (1999).
3. Servicio Geológico Mexicano (SGM). Carta geológico-minera Oaxaca E14-9. Scale 1:250,000, 1 sheet (2000).

4. Bodnar, R. J., Lecumberri-Sanchez, P., Moncada, D., & Steele-MacInnis, M. Fluid Inclusions in Hydrothermal Ore Deposits, in Treatise on Geochemistry, 2nd edition (eds. Holland, H., & Turekian, K. K.) **13**, 119–142 (Oxford, Elsevier Ltd., 2014).
5. Wilkinson, J. J. Fluid Inclusions in Hydrothermal Ore Deposits. Lithos **55**, 229–272; doi:10.1016/B978-0-08-095975-7.01105-0 (2001).
